# Pathophysiological Ionotropic Glutamate Signalling in Neuroinflammatory Disease as a Therapeutic Target

**DOI:** 10.3389/fnins.2021.741280

**Published:** 2021-10-21

**Authors:** Richard Fairless, Hilmar Bading, Ricarda Diem

**Affiliations:** ^1^Department of Neurology, University Clinic Heidelberg, Heidelberg, Germany; ^2^Clinical Cooperation Unit (CCU) Neurooncology, German Cancer Consortium (DKTK), German Cancer Research Center (DKFZ), Heidelberg, Germany; ^3^Department of Neurobiology, Interdisciplinary Center for Neurosciences, Heidelberg University, Heidelberg, Germany

**Keywords:** glutamate excitotoxicity, neuroinflammation, neurodegeneration, Alzheimer’s disease, multiple sclerosis

## Abstract

Glutamate signalling is an essential aspect of neuronal communication involving many different glutamate receptors, and underlies the processes of memory, learning and synaptic plasticity. Despite neuroinflammatory diseases covering a range of maladies with very different biological causes and pathophysiologies, a central role for dysfunctional glutamate signalling is becoming apparent. This is not just restricted to the well-described role of glutamate in mediating neurodegeneration, but also includes a myriad of other influences that glutamate can exert on the vasculature, as well as immune cell and glial regulation, reflecting the ability of neurons to communicate with these compartments in order to couple their activity with neuronal requirements. Here, we discuss the role of pathophysiological glutamate signalling in neuroinflammatory disease, using both multiple sclerosis and Alzheimer’s disease as examples, and how current steps are being made to harness our growing understanding of these processes in the development of neuroprotective strategies. This review focuses in particular on *N*-methyl-D-aspartate (NMDA) and 2-amino-3-(3-hydroxy-5-methylisooxazol-4-yl) propionate (AMPA) type ionotropic glutamate receptors, although metabotropic, G-protein-coupled glutamate receptors may also contribute to neuroinflammatory processes. Given the indispensable roles of glutamate-gated ion channels in synaptic communication, means of pharmacologically distinguishing between physiological and pathophysiological actions of glutamate will be discussed that allow deleterious signalling to be inhibited whilst minimising the disturbance of essential neuronal function.

## Introduction

Neuroinflammation is defined as chronic inflammation of the nervous system, and can occur as a result of infection and autoimmunity, as well as other disorders, such as traumatic brain injury and exposure to toxins, which cause a break-down in the blood-brain barrier (BBB), thus compromising the immune privilege of the CNS. In fact, neuroinflammatory processes are seen in most CNS neurodegenerative disorders, as well as being a common part of the ageing process. The connection between neurodegeneration and neuroinflammation involves complex and intertwined processes whereby inflammation can initiate neuronal degeneration, and conversely neurodegenerative processes can trigger inflammatory mechanisms to promote repair or debris clearance. Since neurodegeneration and neuroinflammation occur in most CNS disorders, in this review we have focussed specifically on two examples. Firstly, multiple sclerosis (MS) which as an autoimmune disease represents inflammation-driven neurodegeneration, and secondly, Alzheimer’s disease (AD) where neurodegeneration is believed to drive the ensuing neuroinflammation. In both cases, however, inadequate therapies are available. For MS, although inflammatory modulating drugs exist, direct neuroprotective strategies are at present still lacking. Similarly, for AD, acetylcholinesterase inhibitors are the main drugs of choice but only serve to treat the symptoms of cognitive impairment, again without achieving neuroprotection. By understanding common elements of these, and other neurological disorders, there may be hope for developing novel neuroprotective strategies. One mechanism common to most neuroinflammatory diseases is glutamate excitotoxicity which, as a convergence point irrespective of disease aetiology, may render it a potential point of therapeutic intervention. Although known for many decades, it has eluded therapeutic modulation due to the essential functions of physiological glutamate signalling. Recent advances in the field have revealed the complexity of glutamate signalling under both physiological and pathophysiological conditions, in both neuronal and non-neuronal cells. Most importantly, an understanding of the different signalling mechanisms involved may allow the targetted disruption of the pathophysiological whilst leaving indispensable physiological functions unaltered.

## Physiological Glutamate Signalling

Glutamate is the main excitatory neurotransmitter of the CNS, with many different glutamate receptors being identified. These range from several families of metabotropic, G-protein-coupled receptors, to the ligand-gated ionotropic *N*-methyl-D-aspartate (NMDA) and 2-amino-3-(3-hydroxy-5-methylisooxazol-4-yl) propionate (AMPA), and kainate receptors. For the scope of this review, although almost every glutamate receptor subtype has been shown to be involved in mediating excitotoxicity in some form (Dong et al., 2009; Crupi et al., 2019), focus will be made on the best understood ionotropic NMDA receptors (NMDARs) and AMPA receptors (AMPARs) due to their recognition as pivotal players in both synaptic signalling and neurodegeneration. AMPARs are the most abundantly expressed receptor in the CNS, and are essential for synaptic transmission and plasticity at many post-synaptic elements (Watson et al., 2017). They are composed of GluA1-GluA4 subunits organised as either homo- or hetero-tetramers, where the subunit composition determines the ionic permeability properties of the channel. NMDARs are similarly widely expressed in the CNS, and are a central element of the processes of synaptic plasticity and memory (Paoletti et al., 2013). They are heteromeric tetramers composed of GluN1 subunits, with additional GluN2 (A-D) or GluN3 (A,B) subunits. Both AMPARs and NMDARs can also work synergistically whereby depolarisation through AMPAR is essential for the expulsion of the positively charged Mg^2+^ ion blocking the pore of NMDAR (Di Maio et al., 2016). Conversely, NMDAR-dependent recruitment of AMPARs is a key process in long term potentiation and the process of memory (Paoletti et al., 2013).

As a result of post-synaptic glutamate receptor activation, the flow of positive ions, particularly Na^+^, into the post-synapse causes depolarisation and the induction of an action potential. However, the influx of Ca^2+^ is also permitted (as well as being amplified through depolarisation-induced opening of L-type voltage-dependent Ca^2+^ channels) where it is involved in regulating diverse neuronal activities through actions as a second messenger. One such activity is the synchronising of mitochondrial activity with that of the neuron such that there is a harmonisation of the energy supply with that needed by the neuron (Kann and Kovacs, 2007). In addition, Ca^2+^ signalling is known to mediate changes in gene transcription, allowing neuronal activity to regulate gene expression. This can be elicited by Ca^2+^-dependent events in both the cytoplasm and the nucleus. In the cytoplasm, Ca^2+^ binding to calmodulin (CaM) leads to the activation of various kinase signalling cascades, involving calmodulin kinase (CaMK) and Ras/mitogen activated protein kinase (Ras/MAPK), causing the phosphorylation and activation of transcription factors such as cyclic AMP response element binding protein (CREB) and nuclear factor kB (NF-kB). In contrast, nuclear Ca^2+^ elevations can lead to modulation of gene transcription through activation of nuclear-located kinases, such as Ca^2+^/calmodulin-dependent protein kinase IV (CaMKIV) (Hardingham et al., 2001), direct Ca^2+^ binding to transcription factor downstream regulatory element antagonistic modulator (DREAM) (Carrion et al., 1999; Ledo et al., 2002), or alternatively through the regulation of histones involved in chromatin structure thus regulating transcription factor accessibility to their target binding sequences [reviewed in Hagenston and Bading (2011)]. Collectively, Ca^2+^ signals elicited by glutamate transmission allows for synaptic strength and energy provision to be regulated by neuronal activity, as well as regulating gene expression programmes involved in dendritic remodelling (Redmond et al., 2002; Mauceri et al., 2011), memory consolidation (Bailey et al., 1996; Hemstedt et al., 2017) neuronal survival/degeneration (Hardingham et al., 2002; Papadia et al., 2005; Lau and Bading, 2009; Zhang et al., 2009; Buchthal et al., 2018), and metabolic plasticity (Bas-Orth et al., 2017).

In addition to their well-described role in neurons, ionotropic glutamate receptors are also found on non-neuronal cells. For example, astrocytes express an array of glutamate receptors, although these differ profoundly depending upon brain region (Verkhratsky and Kirchhoff, 2007; Molders et al., 2018). These allow astrocytes to detect neuronal synaptic activity, and to subsequently respond through the activation of intracellular signalling events, allowing them to generate feedback signals to neurons through the release of gliotransmitters such as ATP (Zhang et al., 2003; Harada et al., 2015). In this manner, astrocytes can shape transmission and regulate synaptic plasticity (Araque et al., 2014). Although astrocytes express mainly metabotropic receptors, they also express AMPARs in most CNS regions (Molders et al., 2018; Ceprian and Fulton, 2019), and also in very low levels NMDARs (Skowronska et al., 2019).

Oligodendrocytes and oligodendrocyte progenitor cells (OPCs) also express AMPARs, which influence OPC migration and development (Fannon et al., 2015; Harlow et al., 2015), proliferation (Gallo et al., 1996; Chen et al., 2000, 2018) and regulate the process of myelination (Kougioumtzidou et al., 2017). OPCs can form synapses with glutamatergic axons (Bergles et al., 2010), and thereby may be influenced by neuronal activity (Barres and Raff, 1993; Gautier et al., 2015). Both oligodendrocytes and OPCs also express NMDARs (Karadottir et al., 2005; De Biase et al., 2011) which can induce intracellular Ca^2+^ transients in mature, myelinating oligodendrocytes (Micu et al., 2006, 2016). This had previously been overlooked, possibly resulting from earlier studies being performed on cultured oligodendrocytes under suboptimal conditions or due to the presence of NMDARs primarily on oligodendrocyte processes rather than their somas (Karadottir and Attwell, 2007).

A further glial cell type expressing ionotropic glutamate receptors are microglia. Microglia from various brain regions express both AMPARs (Noda et al., 2000; Kettenmann et al., 2011) and NMDARs (Newcombe et al., 2008; Kaindl et al., 2012). Specifically, AMPAR stimulation has been demonstrated to regulate microglial motility (Liu et al., 2009; Fontainhas et al., 2011), whereas NMDAR stimulation can induce proinflammatory activities such as the promotion of proliferation and secretion of pro-inflammatory mediators (Raghunatha et al., 2020). It has been suggested that bi-directional crosstalk between neurons and microglia, through mediators such as glutamate, may allow microglia to assess the health status of neighbouring neurons, and similarly for microglial activity to influence basal transmission (Banati, 2002).

In addition to glia, brain endothelial cells have long been reported to express both NMDARs and AMPARs (Krizbai et al., 1998). Endothelial NMDARs are proposed to regulate the reorganisation of the microvasculature (Sailem and Al Haj Zen, 2020) and to coordinate artery dilation (LeMaistre et al., 2012), and thus may couple brain activity with vascular changes to ensure that the blood supply matches energy demand (Hogan-Cann et al., 2019). Similar roles have been suggested for AMPAR (Andras et al., 2007). Moreover, it has also been shown that the NMDAR subunit GluN1 is involved in regulating monocyte migration across the brain endothelial cell barrier (Reijerkerk et al., 2010).

Furthermore, glutamate receptors have also been detected on an array of immune cells. For example, T cells are known to express subunits of both AMPARs (Ganor et al., 2003; Sarchielli et al., 2007), and NMDARs (Boldyrev et al., 2004; Miglio et al., 2005; Affaticati et al., 2011). Upon activation, a change in T cell glutamate receptors occurs with the disappearance of the AMPAR subunit GluA3 (Ganor et al., 2007), and a switch from GluN2B to a combination of GluN2A, B and D NMDAR subunits (Miglio et al., 2005). The function of T cell glutamate receptors may also mediate brain-immune system communication through the myriad of T cell responses that can be elicited depending upon the T cell activation status and the immune environment (Levite, 2008).

## Glutamate Excitotoxicity and Neurodegeneration

It can therefore be seen that glutamate is an essential component of physiological CNS activities through not just mediating neuronal signalling, but also by mediating communication between neurons and other compartments, permitting the coupling of neuronal activity with the activity of their glial support infrastructure, the vasculature as well as the immune system. However, glutamate is also a mediator of neurodegeneration under pathophysiological conditions. Since the 1950s, glutamate has been recognised as a key activator of excitotoxicity whereby excessive glutamate receptor stimulation can result in neuronal death, as was first described in the inner layers of the mouse retina (Lucas and Newhouse, 1957). This was first recognised following intravitreal injection of glutamate, but since then excitotoxic death has been described for most diseases, as well as injuries, affecting the CNS (Doble, 1999; Dong et al., 2009) ranging from neurodegenerative diseases such as AD (Wang and Reddy, 2017), to autoimmune disorders such as MS (Macrez et al., 2016).

Although excessive Na^+^ influx can be toxic to neurons, excitotoxicity appears to arise mainly from a disturbance in the tightly regulated Ca^2+^ homeostasis within the neuron. Under physiological conditions, Ca^2+^ increases arising from influx and intracellular release from Ca^2+^ stores are balanced by several processes. These include Ca^2+^ buffering, by both organelles such as mitochondria and endoplasmic reticulum (Gleichmann and Mattson, 2011) and through interaction with Ca^2+^-binding proteins (Fairless et al., 2019). In addition, Ca^2+^ can be extruded from neurons through the action of exchangers such as the Na^+^/Ca^2+^ exchanger and pumps such as the plasma membrane Ca^2+^ ATPase (PMCA) (Fairless et al., 2014). However, following prolonged or over-stimulation of glutamate receptors, Ca^2+^ increases can exceed the homeostatic capacity of the neuron, resulting in an array of cell death-mediating processes such as the over-activation of Ca^2+^-dependent enzymes, such a calpains and caspases, that either directly damage cellular structures or induce the formation of toxic oxidative free radicals. In addition, the metabolic strain of ATP-dependent Ca^2+^ pump activity, such as the PMCA, can also cause cellular stress, although there is also evidence that metabolic stress can conversely impair PMCA activity resulting in cytotoxicity (Bruce, 2010). Furthermore, Ca^2+^ can be taken up from the cytoplasm by the action of mitochondrial Ca^2+^ transportation mechanisms such as the mitochondrial Ca^2+^ uniporters. Physiologically, mitochondrial uptake and release of Ca^2+^ have been shown to be integrated with cellular Ca^2+^ signalling (Llorente-Folch et al., 2015; Kannurpatti, 2017) and, through coupling of Ca^2+^-sensitive matrix dehydrogenases within the mitochondria, NADH production is increased supporting respiration and energy supply for the neuron (Tarasov et al., 2012). However, excess Ca^2+^ uptake in mitochondria can cause permeabilisation of the outer mitochondrial membrane (OMM) through opening of the permeability transition pore (PTP) (Crompton, 1999; Jia and Du, 2021), a process known as mitochondrial permeability transition, resulting in the release of Ca^2+^ through the pore as well as other matrix components, such as cytochrome c, from the intermembrane space. Opening of the PTP also results in both mitochondrial matrix swelling and a breakdown in the mitochondrial membrane potential (Beutner et al., 2017), which may impair the metabolic state of the cell leading to ROS generation (Qiu et al., 2013). In addition, mitochondrial Ca^2+^ can activate apoptotic pathways through calcineurin activation, which results in, for example, activation via dephosphorylation of the proapoptotic Bcl-2-associated death promoter (Bad) (Wang et al., 1999).

As already mentioned, neuronal gene expression pathways are known to be regulated by intracellular Ca^2+^ allowing cell survival expression programmes to be coupled with synaptic activity. However, under pathophysiological conditions, an opposing gene expression programme can be activated downstream of NMDAR activation. This is primarily achieved through the inactivation and retention of the extracellular signal-regulated kinases (ERK) at the plasma membrane, thus preventing ERK translocation to the nucleus and subsequent interaction with nuclear transcription factor targets (Ivanov et al., 2006; Gao et al., 2010). In addition, a CREB shut-off pathway can also be activated mediated by dephosphorylation of the transcription factor CREB preventing CREB-dependent expression of genes associated with cell survival (Hardingham et al., 2002). Alternative degenerative mechanisms that regulate gene expression include the importation of class IIa histone deacetylases (HCAs) and the proapoptotic transcription factor FoxO3A to the nucleus (Dick and Bading, 2010).

That NMDAR activation can lead to opposing gene expression programmes associated with both cell survival and degeneration has started to be unravelled through the observation that NMDAR receptors can have antithetical functions depending upon their localisation. NMDARs are found in both synaptic and extrasynaptic locations, but despite estimations of the extrasynaptic pool to be around 30–40% of the total NMDAR pool (Harris and Pettit, 2007; Moldavski et al., 2020), the physiological functions of extrasynaptic NMDARs are still being elucidated. They are believed to be activated by either synaptic spill-over or by ectopically released glutamate, such as from astrocytes (Parri et al., 2001; Carmignoto and Fellin, 2006). Thus, they represent an additional pathway by which glia and neurons can communicate. Specifically, glial-derived glutamate has been shown to be involved in neuronal synchronisation, through the evocation of neuronal slow transient currents (Angulo et al., 2004; Carmignoto and Fellin, 2006). Similarly, ambient glutamate acting on extrasynaptic NMDARs can generate a tonic current which can influence neuronal excitability (Wu et al., 2012). However, under pathophysiological conditions, over-stimulation of the different receptor pools can lead to profoundly different results. Whereas cell survival signals are associated with synaptic NMDAR activity, it was shown that only extrasynaptic NMDARs were coupled to excitotoxic events (Hardingham et al., 2002; Hardingham and Bading, 2010). These include the deregulation of transcription, mitochondrial dysfunction through breakdown of the mitochondrial membrane potential and mitochondrial permeability transition, and also a breakdown in structural neuronal integrity, which can be collectively considered as the pathological triad of extrasynaptic NMDA receptor signalling (Bading, 2017). Structural integrity of neurons is lost due to the shut-down of genes involved in the maintenance of complex dendritic architecture and synaptic connectivity, such as brain-derived neurotrophic factor (BDNF) and vascular endothelial growth factor D (VEGFD) (Hardingham et al., 2002; Zhang et al., 2007; Mauceri et al., 2011).

How glutamate elicits different responses via synaptic and extrasynaptic NMDARs is currently unclear, but may involve a differential coupling of NMDARs to mitochondria. Since spines usually lack mitochondria (Li et al., 2004), it may be that only NMDARs in extrasynaptic regions are in close enough proximity to mitochondria (Bading, 2017), resulting in a coupling of Ca^2+^ influx with mitochondrial Ca^2+^ uptake. This may explain the observation that a collapse in the mitochondrial membrane potential was only observed following Ca^2+^ transients initiated by extrasynaptic, but not synaptic, NMDAR activation (Hardingham et al., 2002). An alternative explanation might lie in the composition of the NMDARs themselves. Synaptic and extrasynaptic NMDARs, although vastly similar in terms of their subunit composition, have some differences since the GluN2B subunit is more predominant within extrasynaptic NMDARs, and alternatively synaptic NMDARs have a preference for GluN2A (Paoletti et al., 2013). The relevance of this to excitotoxicity may arise from a direct interaction demonstrated for GluN2B with death-associated protein kinase 1 (DAPK1) (Tu et al., 2010). However, although inhibition of DAPK1-mediated NMDAR phosphorylation by a mimetic peptide was proposed to be neuroprotective in a mouse model of stroke (Tu et al., 2010), a recent study questioned whether DAPK1 was necessary for degeneration to proceed, instead proposing that the peptide could act as a conventional NMDAR channel antagonist (McQueen et al., 2017). More recently, transient receptor potential melastatin subfamily member 4 (TRPM4) has been identified as a novel component of an NMDAR death signalling complex (Yan et al., 2020), which due to the absence of TRPM4 in the synapse (Bayes et al., 2012), is a feature of extrasynaptic NMDARs. Uncoupling of the NMDAR subunits NR2A or NR2B from interacting with TRPM4 was demonstrated to be neuroprotective against NMDAR-mediated excitotoxicity, but most importantly, left the channel function of both NMDAR and TRPM4 unaltered.

## Glutamate Signalling and Neuroinflammatory Disease

Under pathophysiological conditions, in addition to neurons, many other cell types can be adversely affected by glutamate signalling. As outlined above, glutamate is also a regulator of cellular function in glial cells. It is therefore perhaps not surprising that both oligodendrocytes (Yoshioka et al., 1995; Matute et al., 1997) and astrocytes (Chen et al., 2000) have demonstrable vulnerability to glutamate excitotoxicity. Oligodendrocytes and OPCs have primarily been reported to be susceptible to AMPA/kainate receptor-mediated excitotoxicity (Tekkok and Goldberg, 2001; Liu et al., 2002; Stys, 2004). However, consistent with the recent identification of NMDARs on the myelinating processes of mature oligodendrocytes (Karadottir et al., 2005), there is evidence that NMDAR can also mediate oligodendrocyte damage since NMDAR blockade relieved white matter damage following ischaemia (Schabitz et al., 2000; Salter and Fern, 2005; Doyle et al., 2018), and prevented ischaemic ultrastructural myelin damage (Micu et al., 2006).

Endothelial cells derived from the cerebral vasculature have also been reported to be vulnerable to glutamate toxicity (Parfenova et al., 2006), and treatment with either NMDA or glutamate caused a reduction in the brain endothelial barrier integrity *in vitro* as assessed by their electrical resistance (Sharp et al., 2003). Similarly, glutamate also caused a reorganisation of forebrain-derived endothelial cell tight junctions in culture, which could be blocked by NMDAR inhibition (Andras et al., 2007). Thus, it is conceivable that excessive exposure of endothelial cells to glutamate under neuroinflammatory conditions could result in a disturbance in the BBB facilitating the entry of serum-borne components or immune cells. For example, extravasation of the plasma protein fibrinogen has been demonstrated to promote the activation of CNS microglia, leading to axonal damage (Davalos et al., 2012), and thus the process of BBB disruption could further exacerbate neuroinflammation.

Another event coupling neuroinflammatory disease with glutamate excitotoxicity involves changes in glutamate transporter expression. Astrocytes encapsulating neuronal synapses clear glutamate from the extracellular space through the activity of glutamate transporters, such as glutamate transporter 1 (GLT-1) and glutamate aspartate transporter (GLAST). This can provide two levels of control of glutamate signalling: Firstly, they can regulate the temporal duration of the glutamate signal thus shaping the synaptic response; and secondly, through spatial control they restrict glutamate to the vicinity of the synapse, protecting the neuron from glutamate spill-over. Upon glutamate diffusing further afield, there may be greater propensity for extrasynaptic NMDAR activation (Wild et al., 2015). The importance of these transporters can be seen upon their ablation, with the loss of either GLT1 or GLAST resulting in an enhanced susceptibility to injury (Tanaka et al., 1997; Watase et al., 1998). However, under neuroinflammatory conditions, astrocytic expression of glutamate transporters can be modulated. For example, proinflammatory cytokines such as tumour necrosis factor α (TNFα) can induce a decrease in glutamate uptake (Hu et al., 2000), through downregulation of both astrocytic GLT-1 and GLAST expression (Szymocha et al., 2000). Interestingly, glutamate itself can also mediate this function (Takaki et al., 2012), and thus may lead to a vicious cycle of neurodegeneration. The source of both proinflammatory cytokines and glutamate in this context is believed primarily to derive from activated microglia (Hanisch, 2002; Takaki et al., 2012; Welser-Alves and Milner, 2013).

The impact of neuroinflammatory cytokines can also have a more direct impact on neurodegeneration through their exacerbation of glutamate excitotoxicity (Chao and Hu, 1994; Hermann et al., 2001). In the case of TNFα, several mechanisms may contribute to this effect including promoting the release of glutamate or more TNFα from microglia (Kuno et al., 2005), upregulating neuronal expression of AMPAR (Yu et al., 2002) and NMDAR receptor subunits (Wheeler et al., 2009), or even, in addition to effects on glutamate transporters, by triggering astrocytic glutamate release (Bezzi et al., 2001).

**FIGURE 1:**
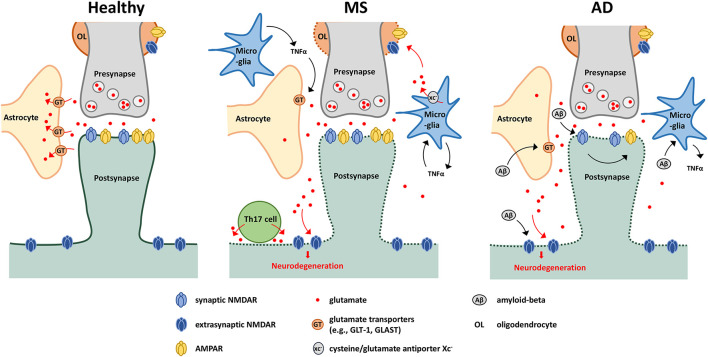
Glutamate-mediated neurodegeneration in MS and AD. Schematic showing possible glutamate-mediated neurodegenerative mechanisms in MS and AD. Under healthy conditions, perisynaptic glutamate levels are maintained through the glutamate cap provided by astrocytes in close proximity of the synapse. Under neuroinflammatory conditions, glutamate spill-over can occur due to downregulation of glutamate transporters, leading to activation of extrasynaptic NMDARs and subsequent neurodegeneration. This downregulation is driven by several effectors including actions of TNFα released by activated microglia or amyloid-beta accumulation. In MS, oligodendrocytes are also sensitive to glutamate excitotoxicity, with glutamate also deriving from activated microglia. Glutamate export is mediated via the cysteine/glutamate antiporter xc^–^, and is driven in part by a TNFα-mediated autocrine mechanism. Th17 cells have also been shown to release glutamate through the formation of an immuno-neuronal synapse. In AD, amyloid beta can stimulate microglia to produce proinflammatory cytokines such as TNFα, and through interaction with NMDARs, drives AMPAR downregulation (affecting synaptic communication), synapse loss and neuronal degeneration.

Collectively, these pathophysiological mechanisms resulting from aberrant glutamate signalling have been implicated in several examples of neuroinflammatory disease, such as MS and AD (Figure 1).

## Pathophysiological Glutamate Signalling in Multiple Sclerosis

Multiple sclerosis is an inflammatory, autoimmune disease which presents in different versions including relapsing-remitting and progressive forms (Dutta and Trapp, 2014). It is characterised by demyelinating lesions, which are typically multifocal areas of inflammation, demyelination and axonal degeneration occurring in the vicinity of BBB breakdown. They can occur in different parts of the CNS resulting in a wide array of symptoms. Despite the cause of the disease being unknown, several risk factors have been identified (Nourbakhsh and Mowry, 2019).

Although predominantly a demyelinating disease, MS can also be considered a neurodegenerative disease since neuronal damage is both a common and early feature of the disease, and accumulation of neurodegeneration may explain the frequent transition from relapsing-remitting MS to a secondary progressive form (Bjartmar and Trapp, 2001; Kuhlmann et al., 2002; De Stefano et al., 2003). Neurodegeneration may arise from the metabolic strain placed on axons transmitting in the absence of myelin (Yin et al., 2016), exposure of demyelinated axons to the action of immune cells, free radicals and proinflammatory cytokines to be found in active regions of the immune response (Trapp and Stys, 2009; Lassmann, 2013), or through glutamate excitotoxicity.

The evidence for glutamate excitotoxicity in MS include elevated glutamate levels in both cerebrospinal fluid (Stover et al., 1997; Sarchielli et al., 2003) and brain lesions (Srinivasan et al., 2005). Furthermore, glutamate transporter expression was shown to be reduced in the vicinity of active MS lesions (Werner et al., 2001; Pitt et al., 2003; Domercq et al., 2005), correlating with areas of axonal and oligodendrocyte damage. Within these lesions, decreased expression occurred mainly in oligodendrocytes, but also in astrocytes though they retained some residual expression (Werner et al., 2001). As noted above, glutamate transporter expression may be influenced by the proinflammatory environment of the MS lesion due to the abundance of cytokines such as TNFα, which can also increase glutaminase activity in microglia (Thomas et al., 2014), further contributing to localised increases in glutamate. In addition to glutamate elevations resulting from decreased clearance or increased microglial activity, immune cells such as T cells may be a further source of excitotoxic glutamate (Liblau et al., 2013; Ganor and Levite, 2014). For example, in the animal model of multiple sclerosis, experimental autoimmune encephalomyelitis (EAE), Th17 cells were reported to be able to induce neuronal degeneration through the establishment of an immuno-neuronal synapse, with neurotoxic effects partially blocked through application of the NMDA receptor blocker, MK-801 (Siffrin et al., 2010).

Several insights into the potential mechanisms of glutamate-mediated neuroinflammation are provided by studies using EAE (Constantinescu et al., 2011). This model recapitulates many of the observations in MS tissue, such as widespread neuronal damage, as well as some of the proposed mechanisms such as decreased expression of glutamate transporters in areas of active inflammation (Ohgoh et al., 2002). Use of this model has confirmed observations from MS that the cysteine/glutamate antiporter x_c_^–^ may have a central role (Pampliega et al., 2011), since pharmacological blockade reduced EAE severity, and genetic ablation rendered mice resistant to disease (Evonuk et al., 2015). X_c_^–^ is known to be a source of toxic glutamate during oxidative stress, and is expressed on many cells within the CNS, such as astrocytes, microglia, Muller glia and immature cortical neurons (Bridges et al., 2012). In EAE, its expression colocalised predominantly with activated microglia (Evonuk et al., 2015), and inhibition was also found to decrease T cell infiltration. Interestingly, NMDAR blockade can also reduce immune cell infiltration in EAE (Bolton and Paul, 1997; Paul and Bolton, 2002; Suhs et al., 2014). Mechanistically, this may reflect the influence of endothelial cell NMDAR activation on the integrity of the BBB (Sharp et al., 2003; Andras et al., 2007; Reijerkerk et al., 2010), as discussed earlier. However, alternative explanations may involve NMDAR activity on other cell types, such as astrocytes which are also involved in maintenance of the BBB (Alvarez et al., 2013), or even NMDAR-independent effects of the blockers, meaning further research is needed in this area. Astrocytic glutamate metabolism might also be impaired in EAE since expression of enzymes such as glutamate dehydrogenase and glutamine synthetase were also down-regulated in astrocytes during EAE (Hardin-Pouzet et al., 1997).

Application of glutamate receptor-specific antagonists in EAE has revealed the contributions of AMPARs and NMDARs to disease pathology. AMPAR blockade ameliorated damage to both oligodendrocytes and axons (Pitt et al., 2000; Smith et al., 2000), as well as protecting synapses and dendritic spines (Centonze et al., 2009). These effects have been attributed primarily to the AMPAR-mediated protection of myelin. NMDAR blockade was similarly neuroprotective demonstrated by the reduction in axonal damage, as well as restricting immune cell infiltration (Paul and Bolton, 2002; Suhs et al., 2014). A further study demonstrated that NMDAR blockade protected neuronal mitochondria and synapses from EAE-associated damage (Grasselli et al., 2013).

Collectively, this illustrates how glutamate can impact several compartments involved in MS/EAE such as the BBB, activation and infiltration of immune cells, glial function, in addition to its influence on neuronal survival. The involvement of these compartments is not just restricted to MS/EAE, but reflects mechanisms involved in other diseases such as AD, and neuroinflammation in general.

## Pathophysiological Glutamate Signalling in Alzheimer’s Disease

Alzheimer’s disease is a progressive neurodegenerative disease characterised by cognitive and functional impairment, and represents one of the most common forms of dementia (Giri et al., 2016). Cognitive impairment is highly correlated with synaptic loss and dysfunction (Scheff et al., 2006), with severe cortical atrophy in later stages due to neuronal loss (Weiner and Frenkel, 2006; Giri et al., 2016). It is characterised by the presence of intracellular neurofibrillary tangles consisting of tau protein deposits, and extracellular amyloid plaques consisting of accumulating amyloid-beta peptides (Weiner and Frenkel, 2006; Wyss-Coray, 2006; Giri et al., 2016). The cause of AD is largely unknown, though impaired clearance of these aggregates appears to be age-related (Vilchez et al., 2014), and an association with several genetic factors, particularly of early-onset AD, have been identified including inherited mutations in amyloid-beta precursor protein (APP), and presenilin genes (PSEN1 and 2) which are involved in amyloid-beta plaque generation (Giri et al., 2016). Furthermore, the onset of cognitive impairment correlates with the emergence of amyloid-beta plaques which follows the appearance of amyloid-beta oligomers (Crimins et al., 2013).

Unsurprisingly due to the presence of degenerating neurons and insoluble plaques and tangles, neuroinflammation is an important aspect of the pathology of AD with identification of various complement proteins, pro-inflammatory cytokines such as interleukin-1, –6, and TNFα, and various other inflammatory proteins (including cyclooxygenases) that are present within the brains of AD patients [reviewed by Akiyama et al. (2000)]. In addition, activated microglia have been detected in brain regions affected by the disease (Akiyama et al., 2000; Cagnin et al., 2001). However, it is debatable whether this inflammation represents a beneficial response which drives the clearance of damaged tissue and protects against further degeneration, or conversely, whether it is a contributor to tissue damage (Akiyama et al., 2000; Wyss-Coray, 2006).

Using animal models of AD, such as APP-overexpressing mice, similar inflammatory pathways appear to be invoked involving both microglial and astrocyte activation, as well as increased production of many of the same inflammatory compounds (Morgan et al., 2005). Using such models, certain factors (including microglial activation and TGFβ expression) have been shown to be relevant to disease progression, whereas others (component C3 of the complement pathway, and IL-1 and –6) did not bear any influence [reviewed by Wyss-Coray (2006)].

A role for glutamate in the pathology of AD is demonstrated since, similar to MS, glutamate clearance may be impaired through reduced expression of cortical glutamate transporters such as GLT-1 (Li et al., 1997; Scott et al., 2011; Hefendehl et al., 2016) and vesicular glutamate transporter 1 (VGLUT1) (Kirvell et al., 2006). It has been proposed that these changes may be mediated by amyloid-beta (Li et al., 2009), as supported in mouse models where GLT-1 expression was reduced in the immediate vicinity of amyloid-beta plaques (Hefendehl et al., 2016). This latter study, using a genetically encoded fluorescence glutamate indicator, was also able to demonstrate alterations in glutamate dynamics around these plaques consistent with impaired clearance, which may underlie the excitotoxic environment in these areas. In addition to impaired clearance, amyloid-beta appears to cause increased extracellular glutamate accumulation through increasing synaptic glutamate release (Brito-Moreira et al., 2011; Zott et al., 2019). Collectively, the effect of amyloid-beta on both glutamate release and subsequent clearance may give rise to neuronal hyperactivity resulting in an impairment in the balance between excitatory and inhibitory neuronal signalling (Lei et al., 2016; Zott et al., 2019), perhaps explaining the increased risk of epilepsy for AD patients (Giorgi et al., 2020).

There is evidence that amyloid-beta can interact with neuronal NMDARs, either directly (Texido et al., 2011; Costa et al., 2012) or via other proteins such as EphB2 (Cisse et al., 2011). The result of these interactions may be synaptic degeneration, since NMDAR inhibition prevented amyloid-beta-mediated changes in synaptic function (Ye et al., 2004; Texido et al., 2011) and structure (Shankar et al., 2007), and ultimately neuronal death (Alberdi et al., 2010). Furthermore, NMDAR-dependent disturbances in synaptic activity and structure were shown to be blocked by NR2B-targetted inhibitors and to correlate with the subcellular localisation of signalling molecules downstream of extrasynaptic NMDARs (Ronicke et al., 2011), supporting a neurotoxic role for NR2B-containing extrasynaptic NMDARs (Paoletti et al., 2013). Similarly, increases in amyloid-beta production were shown to be mediated through extrasynaptic NMDAR activation rather than synaptic NMDARs (Bordji et al., 2010). In fact, amyloid-beta has been reported to preferentially lead to an activation of extrasynaptic NMDARs (Li et al., 2011; Talantova et al., 2013), which was attributed to increased glutamate spill-over from synapses as a result of decreased glutamate clearance (Li et al., 2009). Alternatively, it may be that amyloid-beta-induced decreases in NMDAR expression may affect synaptic and extrasynaptic NMDARs differently, although the use of conditional knock-outs for either NR2A or NR2B appeared to show this not to be the case (Muller et al., 2018). Similarly, the constituent of neurofibrillary tangles, tau, is also involved in extrasynaptic NMDAR activity since NMDA-elicited currents in hippocampal neurons from tau knock-out mice had almost no contribution from extrasynaptic NMDARs (Pallas-Bazarra et al., 2019). The apparent absence of functional extrasynaptic NMDARs in tau knock-out mice was suggested to maybe involve tau association with actin, which may in turn regulate the lateral diffusion of NMDARs from synaptic to extrasynaptic locations. However, the contribution of tau to extrasynaptic NMDAR function in AD is currently unclear.

Amyloid-beta also affects AMPARs by inducing their internalisation away from synapses, leading to synaptic depression and inhibition of long-term potentiation (LTP) (Hsieh et al., 2006). This process appears to be mediated by NMDAR activation, through downstream activation of signalling pathways involving calcineurin, protein phosphatase 1 or the p38 mitogen activated protein kinase (p38 MAPK) signalling pathways (Guntupalli et al., 2016).

Furthermore, in addition to the effects of the soluble amyloid-beta component of APP on glutamatergic receptors, there is also evidence that a further APP cleavage product, APP intracellular domain (AICD), can also affect NMDAR composition and activity (Pousinha et al., 2017). This may have relevance for AD since increased levels of AICD, even in the absence of amyloid-beta, were recently shown to induce AD-like pathological features in mice (Ghosal et al., 2009). Interestingly, the pathophysiological effects of both ACID and amyloid-beta were shown to be inhibited by GluN2B antagonists (Hu et al., 2009; Pousinha et al., 2017), which may be relevant to the excitotoxic role of GluN2B-containing extrasynaptic NMDARs (Paoletti et al., 2013).

Similar to MS, the presence of activated microglia clustered around amyloid plaques leaves open the possibility that microglia may be a further source of elevated glutamate in AD (Mizuno, 2012; Noda, 2016). Although, microglia can support neuroprotection through the clearance of amyloid-beta (Richard et al., 2008; Doi et al., 2009), amyloid-beta-mediated activation of microglia can also cause the production of proinflammatory cytokines such as TNFα and subsequent induction of neuronal death (Combs et al., 2001). The production of microglial glutamate, although induced by APP, is reportedly not induced by amyloid-beta (Barger and Basile, 2001). As reported above, the expression of various glutamate receptors on microglia offers the possibility that neuronal-derived glutamate may also regulate microglial activation in the vicinity of areas of neurodegeneration, and that glutamate receptor antagonism may modulate microglial activity in addition to the benefits from direct neuroprotection.

## Discussion

In both MS and AD, neuroprotective therapies are still lacking. MS patients are routinely treated with immunomodulatory drugs, whilst patients with Alzheimer’s are treated with symptom-targetting therapies, such as those modulating the cholinergic system. This lack of therapeutics has partly arisen due to limitations in the animal models for these diseases, and their relevance for cross-species translation. For example, although EAE has given useful insights into disease mechanisms of MS, and has played an important part in the development of successful therapeutics, it does not reflect all aspects of MS pathophysiology (Constantinescu et al., 2011; Robinson et al., 2014). This is also true of AD animal models, where most models involve overexpression of genes such as APP or amyeloid-beta which result in the formation of amyloid plaques, but in the absence of neurofibrillary tangles (Drummond and Wisniewski, 2017). Similarly, despite the widespread occurrence of amyloid plaques, some of these models have also been lacking in the robust neuronal loss seen in AD (Jankowsky and Zheng, 2017). A further consideration is the translational value of these models between rodents and humans, which has in part been addressed through the increased use of humanised animal models. Several AD models involve the introduction of human mutations associated with familial AD (Jankowsky and Zheng, 2017), though these only account for approximately less than 5% of AD cases (Bekris et al., 2010). In EAE, humanised mice have been used to test the efficacy of antibody therapies raised against human targets (Williams et al., 2018) in order to increase their translational relevance. Thus, the choice of disease model is an important factor in the potential success of identifying suitable therapeutic options. That said, a common key feature of both MS and AD, as well as many of their animal models is glutamate excitotoxicity. It therefore appears that this mechanism acts as a unifying and ultimate pathway in many neurodegenerative diseases, and is therefore an attractive target for therapeutic intervention. In addition, as evidenced above, it is becoming increasingly clear that glutamate signalling has implications for many different compartments of these diseases, such as microglial activity, BBB integrity and integrative cross-talk between glia, neurons and even immune cells, such that modulating glutamate pathophysiological signalling may impact various disease pathomechanisms.

Although AMPARs are implicated in both MS and AD, no major breakthroughs in treatment options have yet emerged, although understanding of the influence of AMPAR subunit composition has fuelled speculation that directed drugs, such as agonists of the plasticity-promoting GluA1-containing AMPARs (Qu et al., 2021), may have therapeutic value. Similarly, selective antagonism of Ca^2+^-permeable AMPARs may be beneficial since they likely mediate AMPAR contributions to neurodegeneration (Weiss, 2011). Ca^2+^ permeability arises from the absence of GluA2 subunits which, due to the addition of a positively charged arginine to the pore lining by RNA editing, prevent Ca^2+^ entry. Alternatively, Ca^2+^ permeable AMPARs may instead contain an unedited GluA2 subunit (Lalanne et al., 2018). However, Ca^2+^-permeable AMPARs also play a key role in plasticity mechanisms (Park et al., 2018), and thus a way to discriminate between “good” and “bad” AMPAR activity is not yet clear.

**FIGURE 2:**
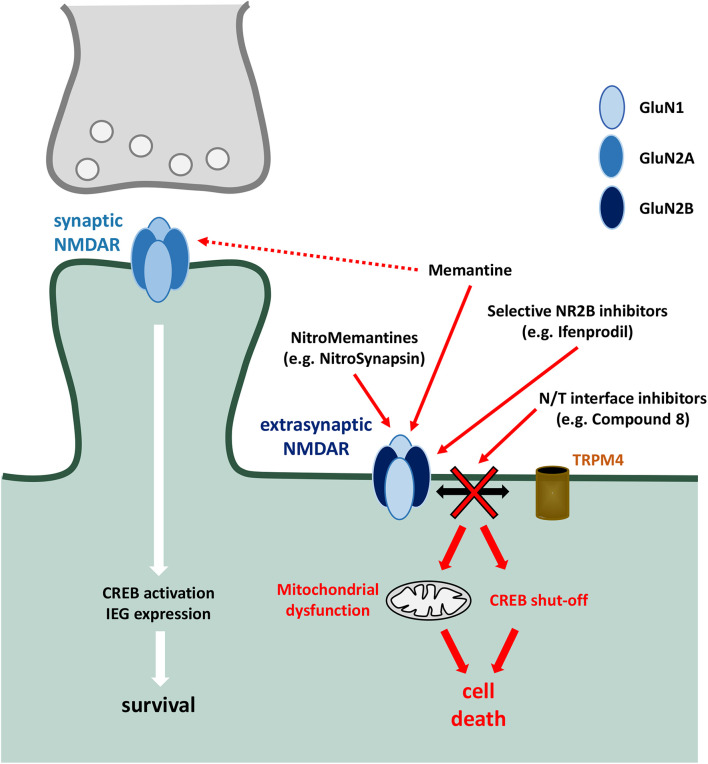
Targetted pharmacological inhibition of extrasynaptic NMDARs. Preferential targetting of extrasynaptic NMDARs is a strategy to inhibit downstream toxic events (including mitochondrial dysfunction, activation of death signalling cascades, CREB dephosphorylation and shut-off, and structural damage), whilst sparing protective mechanisms associated with synaptic NMDAR activity [such as CREB activation and expression of neuroprotective immediate early genes (IEG)]. Memantine shows a preferential inhibitory effect of extrasynaptic NMDARs, which has been harnessed and developed with the NitroMemantine drug series. Selective NR2B inhibitors aim to take advantage of preferential NR2B association within extrasynaptic NMDAR heterotetramers. NDMAR-TRPM4 (N/T) interface inhibitors target the death-promoting complex formed between NMDAR and TRPM4 which, due to the absence of TRPM4 in synapses, mediate the neurodegenerative effects of extrasynaptic NMDAR activation.

Similarly, there is a need to distinguish physiological and pathophysiological NMDAR activity. This has become apparent due to the significant dose-limiting side effects of many NMDAR antagonists (Hewitt, 2000; Lipton, 2004). As such, blockade of NMDAR function with MK-801 can result in the interference of long-term potentiation (Frankiewicz et al., 1996) and the induction of psychosis (Andine et al., 1999). The identification of distinct signalling pathways downstream of synaptic and extrasynaptic NMDARs has revealed strategies to overcome this (Figure 2). For example, the drug memantine has been investigated in depth for its neuroprotective activity whilst leaving physiological NMDAR activity largely intact. This arises partly from its pharmacological profile whereby it is use-dependent (since it requires voltage-dependent expulsion of Mg^2+^ from the NMDAR pore before binding) with fast on/off kinetics (Alam et al., 2017), allowing it to inhibit excessive receptor activity whilst leaving physiological synaptic signalling largely intact. In addition, it has also been reported to specifically block extrasynaptic NMDARs under optimal conditions (Xia et al., 2010), although it reportedly still retains the ability to induce psychotic manifestations, particularly at supratherapeutic doses (Da Re et al., 2015). Furthermore, in addition to direct neuroprotective functions, memantine has also been proposed to act via microglial cells by inhibiting LPS-induced proliferation, perhaps through an NMDAR-independent mechanism (Wu et al., 2009), as well as inhibiting proliferation induced by amyloid-beta (Sestito et al., 2019). Similarly, memantine treatment in animal models of MS reduced immune cell infiltration, suggesting a protective effect on the BBB perhaps via endothelial cell NMDARs, although further research is needed to determine the mechanism (Paul and Bolton, 2002; Suhs et al., 2014).

Indeed, memantine has been trialled in the treatment of both AD and MS. However, in AD, although improvement in patients was seen in terms of cognition and behaviour, the efficacy outcomes reported were modest (Matsunaga et al., 2015; Kishi et al., 2017), with unclear effects on brain volume (Yang et al., 2013). Similarly in MS, trials using memantine have been performed, but no significant effects were seen on either cognitive impairment or disability (Peyro Saint Paul et al., 2016; Turalde et al., 2020). Therefore, the search for novel neuroprotective therapeutic targets and strategies continues.

A further reason to target deleterious extrasynaptic NMDARs, in addition to allowing neuronal transmission via synaptic NMDARs to proceed, would be to avoid inhibition of NMDAR-dependent mechanisms of synaptic plasticity, which in MS have been suggested to underlie compensatory processes (Rossi et al., 2013; Ksiazek-Winiarek et al., 2015). One antagonist which was found to block tonic activity, suggesting its efficacy at extrasynaptic NMDARs, is NitroSynapsin. As a derivative of memantine, NitroSynapsin targets NMDARs whilst simultaneously promoting S-nitrosylation of the receptor, an endogenous mechanism resulting in receptor inhibition (Choi et al., 2000; Takahashi et al., 2007). Interestingly, this method has been shown to have greater efficacy than memantine in protecting synapses in the 3xTg-AD transgenic mouse model (Talantova et al., 2013), whilst also promoting synaptic plasticity using cerebral organoids developed from Alzheimer’s patients (Ghatak et al., 2020).

Another strategy for selective targetting of extrasynaptic NMDARs is the use of GluN2B subunit-selective antagonists, due to the predominant presence of GluN2B on extrasynaptic NMDARs (Paoletti et al., 2013). Several compounds, such as ifenprodil, have been reported to selectively inhibit GluN2B-containing NMDARs, however, there have been concerns regarding their off-target effects which are reportedly similar to the often-abused drugs phencyclidine (PCP) and ketamine (Mony et al., 2009).

A new, alternative strategy, which aims to avoid disturbances to physiological NMDAR activity, derives from the recent identification of a novel NMDAR interaction with TRPM4 as the mediator of death-promoting NMDAR activity (Yan et al., 2020), as described earlier. By disrupting the interface of this interaction, using either small molecules or a viral approach, the deleterious effects of NMDAR over-activation were blocked, whilst leaving the channel properties of both NMDAR and TRPM4 unchanged. Not only that, this approach appears to mainly target extrasynaptic NMDARs, due to the absence of TRPM4 in the synapse (Bayes et al., 2012). The neuroprotective potential of this technique was demonstrated in cell culture models of NMDAR-mediated excitotoxicity and oxygen-glucose deprivation using hippocampal neurons, in addition to *in vivo* models including the middle cerebral artery occlusion stroke model and NMDA-induced retinal ganglion cell degeneration (Yan et al., 2020).

Alternative therapies might act to prevent overactivation of extrasynaptic NMDARs by blocking elevations in synaptic overspill of glutamate, perhaps by preventing decreases in glutamate transporter expression associated with neuroinflammation. Similarly, blocking increases in ambient glutamate through targetting of glutamate release from immune cells, such as T cells and microglia, might be another viable option. Further understanding of the mechanisms regulating these processes would be required in order to facilitate their specific targetting. Whether such approaches would be effective in diseases such as MS and AD remains to be addressed, but collectively, the case can be made that continued investigation of pathogenic glutamatergic signalling, in both neurons and other cells, and the application of these new findings into neuroinflammatory disorders is warranted.

## Author Contributions

RF, HB, and RD contributed to the conception of the manuscript. RF wrote the first draft of the manuscript. All authors contributed to manuscript revision, read and approved the submitted version.

## Conflict of Interest

The authors declare that the research was conducted in the absence of any commercial or financial relationships that could be construed as a potential conflict of interest. The handling editor declared a past co-authorship with one of the author, RD.

## Publisher’s Note

All claims expressed in this article are solely those of the authors and do not necessarily represent those of their affiliated organizations, or those of the publisher, the editors and the reviewers. Any product that may be evaluated in this article, or claim that may be made by its manufacturer, is not guaranteed or endorsed by the publisher.
